# A randomized double-blind trial to measure the absorption characteristics of eicosapentaenoic acid and docosahexaenoic acid rich oil blend with natural lipid-based delivery system

**DOI:** 10.1007/s10068-023-01466-z

**Published:** 2023-12-11

**Authors:** Jennifer Chuang, David Briskey, Jennifer Dang, Arun Rajgopal, Amanda Rao

**Affiliations:** 1 Nutrilite Health Institute, Amway I&S, 5600 Beach Boulevard, Buena Park, CA 90622 USA; 2 RDC Clinical, Brisbane, Australia; 3 School of Human Movement and Nutrition Sciences, The University of Queensland, Brisbane, Australia

**Keywords:** Omega-3 fatty acid, EPA, DHA, Bioavailability, Absorption enhancement

## Abstract

A randomized, double-blinded trial with 65 subjects was conducted to compare the pharmacokinetics between PhytoMarineCelle (PM) that consists of eicosapentaenoic acid and docosahexaenoic acid (EPA + DHA) plus a self-emulsifying drug delivery system (SEDDS), and a standard EPA + DHA ethyl ester (SEE) that does not contain SEDDS. PM showed 1.6-fold greater plasma area under the curve (AUC) than SEE at 300 mg, although no significant difference was observed. PM showed a 3.1 and 3.2-fold (*p* < 0.05) greater plasma AUC than SEE at 500 mg and 1000 mg respectively. The concentration max (Cmax) of EPA + DHA did not change between PM and SEE at 300 mg. Cmax of PM was twofold greater than SEE at 500 mg and 1000 mg respectively. The Cmax of EPA + DHA achieved significant difference (*p* < 0.05) only with the 500 mg dose. The PM formulation increased the bioavailability of EPA + DHA by threefold compared to SEE at 500 and 1000 mg.

## Introduction

Omega-3 fatty acids (n-3 FA) are found within cell membranes and have an important role in maintaining both cellular and overall physical health across several domains such as the cardiovascular, endocrine, immune, and pulmonary systems (Shahidi and Ambigaipalan, 2018). Adequate n-3 FA intake like Eicosapentaenoic Acid (EPA) and Docosahexaenoic Acid (DHA) have been shown to benefit the function of the heart, eyes, and brain. Furthermore, recent findings on DHA and EPA have highlighted other potential benefits such as improving memory (Yurko-Mauro et al., 2015), skin health (Pilkington et al., 2011; Sawada et al., 2020), gut microbiota maintenance (Fu et al., 2021), maintenance of telomere length (Ali et al., 2022; Ogluszka et al., 2020), and even alleviating symptoms in rheumatoid arthritis (Cordingley and Cornish, 2022; Harris et al., 2021; Karr et al., 2011; Troesch et al., 2020; Walchuk and Suh, 2020). EPA and DHA may also play a role in inflammation resolution since they are a source of specialized proresolving mediators and play a major role in the reduction of pro-inflammatory metabolites (Ramirez et al., 2019).

Both EPA and DHA are conditionally essential fatty acids that must be acquired from the diet (Baker et al., 2016; Harris et al., 2021). The Inuit population and their low incidence of acute myocardial infarctions was correlated with the abundance of fish in their diet (Bang and Dyerberg, 1972; Dyerberg and Bang, 1979; Dyerberg et al., 1978). This stand in contrast to the elevated risk for chronic disease (Stark et al., 2016) and low EPA + DHA blood levels (< 6% EPA + DHA of total fatty acids in erythrocyte equivalence) found globally, highlighting the importance of n-3 FA on health and wellbeing.

The convenience, palatability, stability, and the ease of meeting the recommended intake can make supplementation more appealing than eating fish (Maki et al., 2018). Studies have shown that daily intake of more than 300 mg of EPA + DHA is needed to promote optimal health (Schuchardt et al., 2011; Shahidi and Ambigaipalan, 2018; Shahidi and Miraliakbari, 2005). The most common forms of n-3 FA in supplements are in natural triglyceride form or reconstituted ethyl ester form. While fish oil typically contains approximately 30% EPA + DHA (Maki et al., 2018; Schuchardt and Hahn, 2013), the ethyl ester form can be further concentrated to up to 90% EPA + DHA. This advancement in concentration capability reduced the amount of oil needed for a given EPA + DHA dosage and subsequently reduced the number of softgels required to achieve the same EPA + DHA dose. Furthermore, the smaller amount of oil decreased the burden on digestion and may mitigate burp-backs (Maki et al., 2018). For this reason, it is common to see EPA and DHA supplements sold in the ethyl ester form worldwide (Schuchardt et al., 2011).

The absorption of n-3 FA like EPA + DHA is sensitive to the composition of the foods that are co-consumed. The lowest absorption occurs when EPA + DHA is taken on an empty stomach while the highest absorption occurs when EPA + DHA is taken with a fatty meal (Ludwig et al., 2018; Maki and Dicklin, 2019). There is a need to develop an EPA + DHA ethyl ester blend with an improved bioavailability regardless.

There are various approaches to improve the solubility and bioavailability of poorly water-soluble compounds (Amara et al., 2019; Bremmell et al., 2020; Kommuru et al., 2001; Maki et al., 2018; Qin et al., 2017). Clinical studies utilized emulsification delivery system has shown increased absorption of EPA and DHA (Bremmell et al., 2020; Maki and Dicklin, 2019; Maki et al., 2018; Qin et al., 2017; West et al., 2018). In this study, plant-based oils are used to construct a self-emulsifying drug delivery system (SEDDS) to enhance the absorption of EPA + DHA ethyl esters. This study aimed to compare the absorption of EE EPA + DHA in the PhytoMarineCelle (PM) to the absorption of EE EPA + DHA in a standard blend (SEE) that does not contain SEDDS at 300 mg, 500 mg, and 1000 mg doses, in healthy participants, over a 24-h period.

## Materials and methods

### Dose characteristics

Both PM and SEE consist of EPA + DHA at 60:40 ratio in ethyl ester form, sourced from Anchovy, Sardines and Mackerel as well as oil from chia seeds and sunflower seeds. The SEDDS formulation consists of polyglycerol ester of fatty acid, lecithin, oil from lime, coconut, and olive as well as mixed d-Tocopherol. PM contains all the same ingredients as the SEE plus the ingredients that make up SEDDS.

### Clinical design

**Fig. 1:**
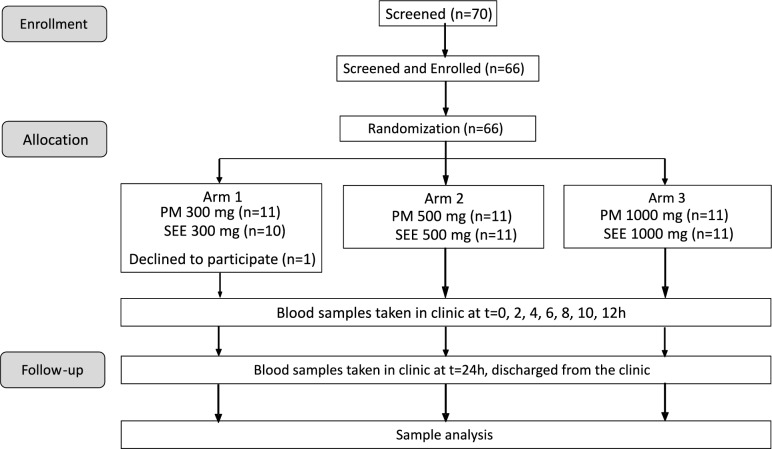
Clinical design schematic; PM = PhytoMarineCelle formula, SEE: Standard EE EPA + DHA, AUC: Area under the curve, Cmax = Peak plasma concentration, Tmax = Time taken to reach Cmax

A randomized, double-blinded study (Fig. 1) was conducted to evaluate the absorption of PM and SEE at 3 doses (300 mg, 500 mg, and 1000 mg). The study was powered to detect a 20% difference in AUC (e.g., 1000 ± 120 vs 800 ± 120) between groups with 95% power and error probability 5%. Based on these parameters, the effect size was 1.667 with 9 subjects required per group. Allowing for dropouts, up to 11 participants per group were recruited. Following enrollment, participants were randomly allocated into the PM or SEE groups in one of 3 dosage arms by using random allocation software (sealedenvelope.com). There are 11 participants per group with an allocation ratio of 1:1. Arm 1 received 300 mg of either PM or SEE formulation. Arm 2 received 500 mg of either PM or SEE formulation. Arm 3 received 1000 mg of either PM or SEE formulation. Only dose-matching pairs were compared. The DHA and EPA composition for both formulations as well as the number of soft-gel capsules of each arm is listed in Table 1. All trial participants and investigators were blinded to the allocations until analysis (including statistics) of all plasma samples had been completed. Participants were required to avoid foods containing omega-3 fatty acids (a list was provided) from 48 h prior to testing until after the collection of the final blood sample was completed.

**Table 1:** Omega-3 fatty acid composition in the PhytoMarineCelle (PM) and EE EPA + DHA (SEE) groups

Arm	Softgel capsules (*n*)	EPA (mg)	DHA (mg)	Total (mg)
1	1	172	128	300
2	2	286	214	500
3	2	572	428	1000

### Inclusion and exclusion criteria

All participants provided written informed consent and were screened against the inclusion and exclusion criteria. Participants were healthy men and women aged over 18 years old, body mass index (BMI) which is a measure of body fat measured by dividing weight of the person in kilogram by square of that person’s height in meters was between 18.5—29.9 kg/m^2^ with no history or evidence of clinically significant medical conditions. These included, but were not limited to; cardiovascular, neurological, psychiatric, renal, immunological, endocrine, and hematological conditions, no allergy to fish or shellfish, no concomitant use of anticoagulant drugs, not pregnant, not a regular user of supplements containing n-3 FA, not taking any kind of prescription medication except the contraceptive pill, no history of substance abuse including alcohol, and not currently a smoker.

### Ethics and clinical registration

This trial was conducted in compliance with the current International Conference on Harmonization Guidelines for Good Clinical Practice, and the Therapeutic Goods Administration. Data recorded and maintained for this study will be controlled in accordance with the National Privacy Principles and Privacy Act 1988. The study was approved by the Human Research and Ethics Committee at the University of Queensland, St. Lucia, Brisbane, Queensland, Australia, approval number 2018002290. The clinical trial was registered on ANZCTR register and the trial registration number is ACTRN 12619000953134.

### Dosing and sample collection

Participants were required to avoid foods containing n-3 FA, mainly EPA and DHA (a list was provided) from 48 h prior to testing until the collection of the final blood sample was complete. Following consent, participants arrived at the clinic having fasted overnight and had a cannula inserted into a vein in the antecubital fossa. Participants consumed the study dose after collecting the baseline blood sample. Detailed EPA and DHA composition in each dose is listed in Table 1. All blood samples were collected into vacutainers containing lithium heparin (BD, New Jersey). The n-3 FA pharmacokinetics were determined from blood samples taken prior to dosing (t = 0), and 2, 4, 6, 8, 10, 12, and 24-h post supplementation. Collected blood samples were immediately centrifuged at 4 ◦C for 10 min (2800 RPM). Once spun, aliquots of plasma were stored at -80 ◦C for analysis.

During the initial 12 h of testing, participants remained at the clinic and were provided with standardized meals containing approximately 25% fat. Breakfast was served after the t = 0 blood draw and within 15-min following the consumption of the study dose while lunch was served after 4h blood draw, and dinner was served after 10h blood draw. The diet does not contain EPA or DHA, and only negligible amounts of ALA. Following the 12-h blood draw, participants had their cannula removed and were discharged from the clinic. Upon discharge, participants were provided with a list of foods to avoid (containing n-3 FA). Participants were asked to return the following morning to assess dietary compliance and provide a 24-h sample for fasting blood draw. Throughout the study, participants were monitored for adverse effects to the supplements.

### Primary and secondary outcome

In this study, the pharmacokinetic parameters measured included are AUC_0-24_, C_*max*_, T_*max*_ and C_*max*_. The primary outcome of the study was to compare the difference in the area under the n-3 FA plasma concentration curve within 24-h (AUC_0−24_) between the PM and SEE formulations. The secondary outcome was to compare the n-3 FA maximum concentration (C_*max*_) as well as the time to reach the maximum concentration (T_*max*_) between PM and SEE groups. Parameters such as gastrointestinal tolerability and reflux were also evaluated to assess tolerability.

### Analysis of omega-3 fatty acids from plasma

Plasma n-3 FA concentrations were analyzed by gas chromatography (GC) with flame ionization detection. Plasma was transferred to a screwcap glass vial which contained heptadecanoic acid as an internal standard (C17:0 FFA) (Sigma-Aldrich, St. Louis, MO) and the methylation reagent (methanol containing 14% boron trifluoride, toluene, methanol; 35:30:35 v/v/v; Sigma-Aldrich, St. Louis, MO) was added as modified Morris and Smith reaction. The vial was briefly vortex mixed and heated in a hot bath at 100°C for 45 min. After cooling, hexane (EMD Chemicals, USA) and HPLC grade water were added, the tubes were recapped, vortex mixed and centrifuged to separate layers. An aliquot of the hexane layer was transferred to a GC vial. GC was carried out using a GC-2030 Gas Chromatograph (Shimadzu Corporation, Columbia, MD) equipped with a SP-2560, 100-m fused silica capillary column (0.25 mm internal diameter, 0.2 µm film thickness; Supelco, Bellefonte, PA).

### Statistical analysis

Plasma concentrations were calculated as change from baseline to account for the endogenous EPA and DHA. The AUC_0−24_, Cmax and Tmax calculations were performed on baseline corrected data for each individual and then calculated and reported as geometric mean ± geometric standard deviation (SD). The area under the curve values (AUC_0−24_), maximum concentration (Cmax), and time to maximum concentration (Tmax) for each participant were calculated in GraphPad Prism 7 using the trapezoidal model for 0–24 h. Statistical significance for AUC and Cmax was conducted using Mann–Whitney analysis carried out via GraphPad Prism 7.

## Results and discussion

### Participant demographics

A total of 27 men and 39 women gave consent and were included in the study. There were no adverse events reported. One participant declined to participate, and a total of 65 participants with an average age of 33.3 ± 6.7 and 32.4 ± 7.6 years in the PM and SEE groups respectively completed the study. Baseline EPA and DHA blood concentrations as well as other participant demographics are in Table 2. No significant differences were found between groups at baseline in terms of age, BMI, EPA and DHA concentrations.

**Table 2:** Participant demographics and baseline blood concentration of Omega-3 fatty acid

Dose (mg)	Age (years)	BMI (kg/m^2^)	Male (*n*)	Female (*n*)	Baseline EPA + DHA (ug/ml)
300 SEE	32.2 ± 8.3	24.7 ± 2.9	6	5	50.0 ± 1.9
300 PM	34.9 ± 5.5	25.3 ± 1.9	3	7	39.9 ± 3.1
500 SEE	33.0 ± 6.9	24.4 ± 2.9	4	7	50.8 ± 2.6
500 PM	32.6 ± 7.2	23.7 ± 2.8	4	7	46.7 ± 1.8
1000 SEE	32.2 ± 7.8	24.5 ± 3.1	4	7	43.3 ± 2.2
1000 PM	32.5 ± 7.3	24.2 ± 2.8	5	6	42.8 ± 2.1

Age and BMI are presented as mean ± SD, the sum of EPA and DHA are presented as geometric mean ± geometric SD

*BMI* body mass index, *EPA* eicosapentaenoic acid, *DHA* docosahexaenoic acid, *SEE* Standard EE EPA + DHA, *PM* PhytoMarineCelle

### Change in EPA and DHA concentrations

Comparing PM with SEE, both EPA and DHA showed > 2-foldchange in AUC_0−24_ at 500 and 1000 mg dose (Table 3). The AUC_0-24_ of EPA was 2.9- and 3.6-fold higher (*p* < 0.05) in the PM group than the SEE group at 500 and 1000 mg dose respectively. The AUC_0-24_ of DHA was 3.3- and 2.7-fold higher in the PM group than the SEE group at 500 and 1000 mg dose respectively. Comparing PM with SEE, both EPA and DHA showed about a twofold change in Cmax at 500 and 1000 mg dose. The Cmax in the PM group increased as the dose increased (EPA increased from 4.45 to 11.27 µg/ml; DHA increased from 4.62 to10.85 µg/ml) whereas the SEE group stayed relatively unchanged (EPA changed from 4.23 to 5.49 µg/ml; DHA changed from 4.68 to5.29 µg/ml). There were no significant changes in the Tmax between PM and SEE at any dose of EPA and DHA. While no significant effect was observed between the SEE and PM formula at 300 mg, the data trended towards significance. A couple factors could have contributed to the variance in the data. The baseline EPA + DHA values in the 300 mg SEE group was slightly higher than the PM group (50 and 39.9 µg/ml respectively) and could have contributed to the increased data variability. Another possibility could be the dose at 300 mg did not produce a large enough change beyond the existing endogenous EPA and DHA. A crossover clinical design may help elucidate the difference at 300 mg. EPA showed greater AUC_0-24_ compared to DHA. The reasons for this are not completely known but may be due to several possibilities. There is slightly higher EPA than DHA in the formula and previous studies have observed that DHA may have reduced absorption through the gastrointestinal epithelium compared to EPA because it is susceptible to oxidation (Dasilva et al., 2018). Additionally, DHA is readily metabolized into a number of potentially bioactive compounds and other fatty acids (Kuda, 2017). Together, these may account for the lower AUC_0-24_ in DHA compared to EPA.

**Table 3:** Summary of EPA, DHA, and EPA + DHA combined pharmacokinetic parameters by dose and formulation

Dose	Standard EE EPA + DHA (SEE)			PhytoMarineCelle (PM)			Fold change	
	AUC (0–24)	Tmax (h)	Cmax (ug/ml)	AUC (0–24)	Tmax (h)	Cmax (ug/ml)	AUC	Cmax
EPA								
300 mg	36.03 ± 4.15	8.91 ± 1.93	4.23 ± 1.94	40.18 ± 2.87	9.55 ± 2.33	4.45 ± 1.91	1.1	1.05
500 mg	36.68 ± 3.20	7.03 ± 2.18	4.78 ± 1.94	106.62 ± 1.92*	8.21 ± 1.89	9.64 ± 1.56*	2.9*	2.0*
1000 mg	40.86 ± 4.12	8.58 ± 1.59	5.49 ± 2.69	148.02 ± 1.53*	9.02 ± 1.77	11.27 ± 1.42*	3.6*	2.1*
DHA								
300 mg	21.00 ± 2.40	8.91 ± 2.55	4.68 ± 1.92	48.65 ± 2.90	15.30 ± 15.3	4.62 ± 2.31	2.3	0.98
500 mg	23.76 ± 4.41	5.04 ± 2.44	4.30 ± 2.41	79.53 ± 2.32	9.10 ± 1.85	8.72 ± 1.90	3.3	2.0
1000 mg	31.31 ± 6.11	6.53 ± 2.02	5.29 ± 3.56	85.40 ± 3.39	7.21 ± 2.20	10.85 ± 1.86	2.7	2.1
EPA + DHA								
300 mg	57.03 ± 3.07	8.91 ± 1.47	8.91 ± 1.99	88.83 ± 2.3	12.43 ± 1.87	9.08 ± 2.26	1.6	1.0
500 mg	60.44 ± 3.35	12.07 ± 1.81	9.08 ± 2.01	186 ± 1.67*	8.66 ± 1.89	18.36 ± 1.74*	3.1*	2.0*
1000 mg	72.16 ± 3.35	7.55 ± 2.13	10.78 ± 2.68	233.4 ± 1.69*	8.12 ± 1.68	22.12 ± 1.52	3.2*	2.1

Area under the curve, Cmax = Peak plasma concentration, Tmax = Time taken to reach Cmax

AUC(0–24) as geometric mean ± geometric SD, Tmax (h) mean ± SD, Cmax (*μ*g/ml) as geometric mean ± geometric SD

*Significant difference *p* < 0.05

### Comparison of total EPA + DHA concentration

**Fig. 2:**
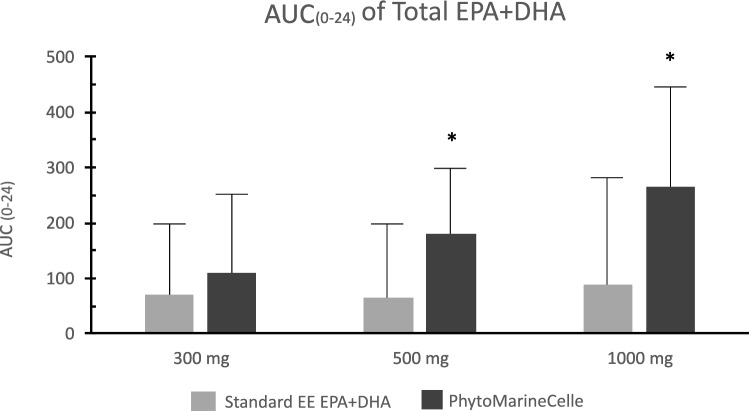
The area under the curve of total EPA + DHA over 24 h after a single oral dose of EE EPA + DHA or PhytoMarineCelle at 300 mg, 500 mg and 1000 mg. Data shown in geometric mean and geometric SD. The PhytoMarineCelle formulation at 500 and 1000 mg were significantly different than the EE EPA + DHA formulation. Gray- Standard EE EPA + DHA; black- PhytoMarineCelle; *p < 0.05

**Fig. 3:**
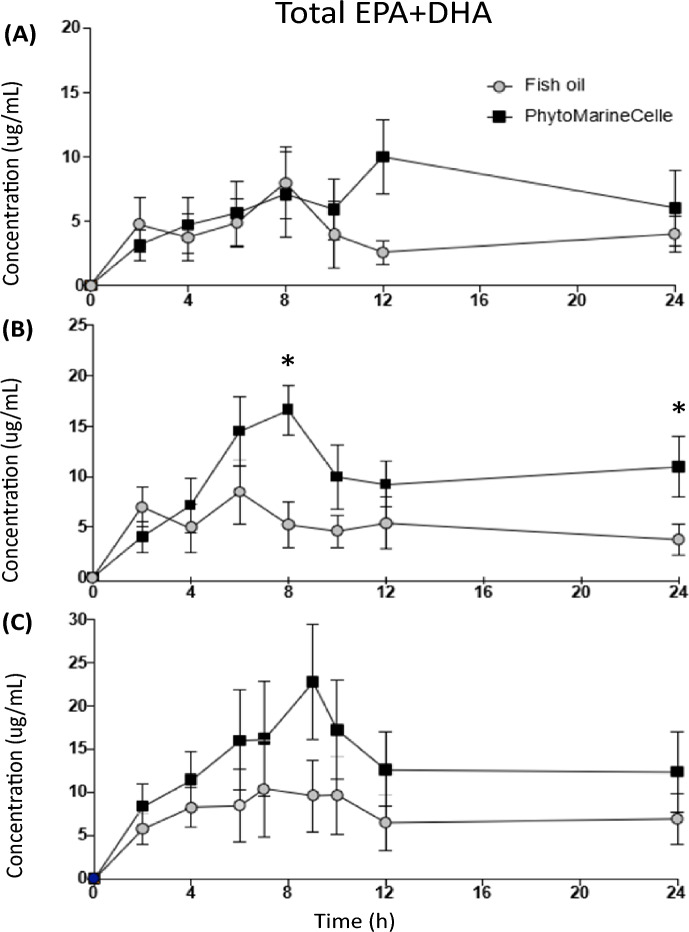
Baseline corrected plasma concentration of total EPA + DHA (average ± SE) in plasma after a single dose of PhytoMarineCelle or EE EPA + DHA. (A) 300 mg of EPA + DHA (B) 500 mg of EPA + DHA (C) 1000 mg of EPA + DHA. *p < 0.5

Comparing PM with SEE, the AUC_0*−*24_ of the total EPA + DHA showed a threefold change at 500 and 1000 mg dose (Table 3). Figure 2 shows the AUC_0*−*24_ of total EPA + DHA in the SEE and PM groups at all three doses. A significant difference in the AUC was observed between the PM and SEE groups at the 500 and 1000 mg doses (*p* < 0.05). Furthermore, the plasma concentration of total EPA + DHA over time showed that the PM formula maintained a higher concentration than the SEE formula throughout most time points at 500 and 1000 mg doses (Fig. 3).

### Comparison of EPA and DHA C_max_ values

The Cmax in total EPA + DHA for 500 mg dose was significantly higher (~ twofold; *p* < 0.05) in the PM than in the SEE formulation. The 100 mg dose came close to achieving significance (*p* = 0.07) while the 300 mg dose did not achieve significance (Fig. 3).

The data from this study showed that SEDDS technology improved the EPA + DHA absorption (AUC_0−24_) by ~ threefold and Cmax by twofold when taken at 500 or 1000 mg dose.

The absorption of omega-3 fatty acids is known to have high interpersonal variability and its cause has not been fully elucidated. Whether it is the gut microbiome, genetic variance or other lifestyle factors that influence response to supplementation, the use of natural oils and surfactants in PhytoMarineCelle is able to bypass these issues and increase the absorption rate by three-fold as seen in Table 3. The geometric standard deviation of the AUC and Cmax associated with PhytoMarineCelle reflects a lower interpersonal variation when compared with the standard EE EPA + DHA, and this difference becomes more pronounced as the dose increases. In addition, experiential factors such as the reduction of burp-back and smaller soft gel size are benefits that a consumer can appreciate. Considering these factors of improving absorption, improving supplementation experience the benefits of PhytoMarineCelle formulation outweighs the cost and resources associated with processing.

### Safety parameters

There were no adverse events reported during this study. The ingredients used and the dose administered in this study are generally regarded as safe and were well tolerated.

The primary limitation of this study was the interpersonal variability between participants which contributed to the data having a wide range. Although the individuals were healthy and not statistically different in age and weight, the observed variability is attributed to the differences between individual’s metabolic rate. The unique metabolic characteristics of EPA and DHA in terms of its conversion and clearance rates could impact AUC calculations. Future studies may benefit from a crossover design to help reduce interpersonal variability. Lastly in this study plasma concentration of EPA and DHA in serum is used as an indicator of absorption. This surrogate method of evaluating absorption does not account for contributions of EPA and DHA from other sources like the liver, adipose tissue, or retro conversion between omega-3 fatty acids.

In conclusion, this randomized, double-blind clinical trial was able to show a threefold higher bioavailability of total EPA + DHA in PM compared to the SEE.

## References

[CR1] Ali S, Scapagnini G, Davinelli S (2022). Effect of omega-3 fatty acids on the telomere length: a mini meta-analysis of clinical trials. Biomolecular Concepts.

[CR2] Amara S, Bourlieu C, Humbert L, Rainteau D, Carriere F (2019). Variations in gastrointestinal lipases, pH and bile acid levels with food intake, age and diseases: possible impact on oral lipid-based drug delivery systems. Advanced Drug Delivery Reviews.

[CR3] Baker EJ, Miles EA, Burdge GC, Yaqoob P, Calder PC (2016). Metabolism and functional effects of plant-derived omega-3 fatty acids in humans. Progress in Lipid Research.

[CR4] Bang HO, Dyerberg J (1972). Plasma lipids and lipoproteins in Greenlandic west coast Eskimos. Acta Medica Scandinavica.

[CR5] Bremmell KE, Briskey D, Meola TR, Mallard A, Prestidge CA, Rao A (2020). A self-emulsifying omega-3 ethyl ester formulation (AquaCelle) significantly improves eicosapentaenoic and docosahexaenoic acid bioavailability in healthy adults. European Journal of Nutrition.

[CR6] Cordingley DM, Cornish SM (2022). Omega-3 fatty acids for the management of osteoarthritis: a narrative review. Nutrients.

[CR7] Dasilva G, Boller M, Medina I, Storch J (2018). Relative levels of dietary EPA and DHA impact gastric oxidation and essential fatty acid uptake. The Journal of Nutritional Biochemistry.

[CR8] Dyerberg J, Bang HO (1979). Haemostatic function and platelet polyunsaturated fatty acids in Eskimos. The Lancet.

[CR9] Dyerberg J, Bang HO, Stoffersen E, Moncada S, Vane JR (1978). Eicosapentaenoic acid and prevention of thrombosis and atherosclerosis?. The Lancet.

[CR10] Fu Y, Wang Y, Gao H, Li D, Jiang R, Ge L (2021). Associations among dietary omega-3 polyunsaturated fatty acids, the gut microbiota, and intestinal immunity. Mediators Inflammation.

[CR11] Harris WS, Tintle NL, Imamura F, Qian F, Korat AVA, Marklund M (2021). Blood n-3 fatty acid levels and total and cause-specific mortality from 17 prospective studies. Nature Communications.

[CR12] Karr JE, Alexander JE, Winningham RG (2011). Omega-3 polyunsaturated fatty acids and cognition throughout the lifespan: a review. Nutritional Neuroscience.

[CR13] Kommuru TR, Gurley B, Khan MA, Reddy IK (2001). Self-emulsifying drug delivery systems (SEDDS) of coenzyme Q10: formulation development and bioavailability assessment. International Journal of Pharmaceutics.

[CR14] Kuda O (2017). Bioactive metabolites of docosahexaenoic acid. Biochimie.

[CR15] Ludwig DS, Willett WC, Volek JS, Neuhouser ML (2018). Dietary fat: from foe to friend?. Science.

[CR16] Maki KC, Dicklin MR (2019). Strategies to improve bioavailability of omega-3 fatty acids from ethyl ester concentrates. Current Opinion in Clinical Nutritions & Metabolic Care.

[CR17] Maki KC, Palacios OM, Buggia MA, Trivedi R, Dicklin MR, Maki CE (2018). Effects of a self-micro-emulsifying delivery system formulation versus a standard omega-3 acid ethyl ester product on the bioavailability of eicosapentaenoic acid and docosahexaenoic acid: a study in healthy men and women in a fasted state. Clinical Therapeutics.

[CR18] Ogluszka M, Te Pas MFW, Polawska E, Nawrocka A, Stepanow K, Pierzchala M (2020). Omega-3 alpha-linolenic fatty acid affects the level of telomere binding protein trf1 in porcine skeletal muscle. Animals (basel).

[CR19] Pilkington SM, Watson RE, Nicolaou A, Rhodes LE (2011). Omega-3 polyunsaturated fatty acids: photoprotective macronutrients. Experimental Dermatology.

[CR20] Qin Y, Nyheim H, Haram EM, Moritz JM, Hustvedt SO (2017). A novel self-micro-emulsifying delivery system (SMEDS) formulation significantly improves the fasting absorption of EPA and DHA from a single dose of an omega-3 ethyl ester concentrate. Lipids Health and Disease.

[CR21] Ramirez JL, Gasper WJ, Khetani SA, Zahner GJ, Hills NK, Mitchell PT (2019). Fish oil increases specialized pro-resolving lipid mediators in PAD (The OMEGA-PAD II trial). Journal of Surgical Research.

[CR22] Sawada Y, Saito-Sasaki N, Nakamura M (2020). Omega 3 fatty acid and skin diseases. Front Immunol.

[CR23] Schuchardt JP, Hahn A (2013). Bioavailability of long-chain omega-3 fatty acids. Prostaglandins Leukot Essent Fatty Acids.

[CR24] Schuchardt JP, Schneider I, Meyer H, Neubronner J, von Schacky C, Hahn A (2011). Incorporation of EPA and DHA into plasma phospholipids in response to different omega-3 fatty acid formulations–a comparative bioavailability study of fish oil vs krill oil. Lipids Health and Disease.

[CR25] Shahidi F, Ambigaipalan P (2018). Omega-3 polyunsaturated fatty acids and their health benefits. Annu Rev Food Sci Technol.

[CR26] Shahidi F, Miraliakbari H (2005). Omega-3 fatty acids in health and disease: part 2–health effects of omega-3 fatty acids in autoimmune diseases, mental health, and gene expression. Journal of Medicinal Food.

[CR27] Stark KD, Van Elswyk ME, Higgins MR, Weatherford CA, Salem N (2016). Global survey of the omega-3 fatty acids, docosahexaenoic acid and eicosapentaenoic acid in the blood stream of healthy adults. Progress in Lipid Research.

[CR28] Troesch B, Eggersdorfer M, Laviano A, Rolland Y, Smith AD, Warnke I (2020). Expert opinion on benefits of long-chain omega-3 fatty acids (DHA and EPA) in aging and clinical nutrition. Nutrients.

[CR29] Walchuk C, Suh M (2020). Nutrition and the aging retina: a comprehensive review of the relationship between nutrients and their role in age-related macular degeneration and retina disease prevention. Advanced Food and Nutrition Research.

[CR30] West AL, Kindberg GM, Hustvedt SO, Calder PC (2018). A novel self-micro-emulsifying delivery system enhances enrichment of eicosapentaenoic acid and docosahexaenoic acid after single and repeated dosing in healthy adults in a randomized trial. J Nutr.

[CR31] Yurko-Mauro K, Alexander DD, Van Elswyk ME (2015). Docosahexaenoic acid and adult memory: a systematic review and meta-analysis. PLoS One.

